# Pangenome Architecture and Accessory Gene-Driven Population Structure of *Staphylococcus aureus* Revealed by a Hospital-Adjacent Environmental Isolate

**DOI:** 10.3390/microorganisms14040938

**Published:** 2026-04-21

**Authors:** Wellington Francisco Rodrigues, Laise Mazurek, Renata Botelho Miguel, Geovana Pina Vilela, Amanda Bertinetti Tres, Sabrina Martins Calegari, Ferdinando Agostinho, Jamil Miguel-Neto, Melissa Carvalho Martins-de-Abreu, Karen M. Wagner, Christophe Morisseau, Carlos Ueira-Vieira, Mariana Santos Cardoso, Aristóteles Góes-Neto, Carlo José Freire Oliveira, Siomar de Castro Soares, Camila Botelho Miguel

**Affiliations:** 1 Department of Entomology and Nematology and UC Davis Comprehensive Cancer Center, University of California, Davis, CA 95616, USAchmorisseau@ucdavis.edu (C.M.); 2 Laboratory of Genetics, Biotechnology Institute, Federal University of Uberlândia, Uberlandia 38400-902, MG, Brazil; laise@unifimes.edu.br (L.M.); renatabotelhonutri@gmail.com (R.B.M.); ueira@ufu.br (C.U.-V.); 3 Multidisciplinary Laboratory of Scientific Evidence, University Center of Mineiros (Unifimes), Mineiros 75833-130, GO, Brazil; gepinavilela@academico.unifimes.edu.br (G.P.V.); amandabertinetti@academico.unifimes.edu.br (A.B.T.); sabrinacalegri@academico.unifimes.edu.br (S.M.C.); jamil@unifimes.edu.br (J.M.-N.); dramelissa@unifimes.edu.br (M.C.M.-d.-A.); camilabmiguel@hotmail.com (C.B.M.); 4 Molecular and Computational Biology of Fungi Laboratory, Department of Microbiology, Instituto de Ciências Biológicas, Universidade Federal de Minas Gerais, Belo Horizonte 31270-901, MG, Brazil; marianascardoso@yahoo.com.br (M.S.C.); arigoesneto@gmail.com (A.G.-N.); 5 Postgraduate Program in Tropical Medicine and Infectious Diseases, Federal University of the Triângulo Mineiro, UFTM, Uberaba 38025-180, MG, Brazil; siomar.soares@uftm.edu.br; 6 Faculty of Physiotherapy, University of Rio Verde, UniRv, Rio Verde 75901-970, GO, Brazil; ferdinando@unirv.edu.br

**Keywords:** *Staphylococcus aureus*, pangenome analysis, comparative genomics, environmental isolate, One Health

## Abstract

*Staphylococcus aureus* is a globally distributed bacterium that spans interconnected human, animal, and environmental niches and is a major driver of antimicrobial resistance. Environmental and wildlife-associated isolates from hospital-surrounding settings remain underrepresented in comparative genomic studies. To address this gap, we integrated a newly sequenced environmental isolate recovered from pigeon fecal samples collected around a hospital into a standardized pangenome framework composed of 99 reproducibly selected RefSeq genomes plus the environmental isolate S_S3. Using uniform genome annotation and orthologous gene family clustering, we identified an open pangenome of 8366 gene families (Heaps’ law γ = 0.275), consistent with the high genomic plasticity previously reported for *S. aureus*. The core genome stabilized at approximately 1757 genes, including 1651 genes conserved across all genomes. Gene frequency spectra showed a dominant cloud genome and a structured shell fraction contributing to interstrain differentiation. Jaccard-based gene content similarity resolved clusters shaped mainly by accessory gene composition. The environmental isolate retained the complete core genome, carried only 15 isolate-specific gene families (0.18% of the pangenome), and clustered within an established lineage. Its unique content included a lincosamide resistance-associated locus and efeB, a gene potentially related to heme or iron metabolism and oxidative stress response. These findings highlight a conserved genomic backbone over a dynamic accessory reservoir and support One Health genomic surveillance that includes wildlife-associated niches, while indicating that the environmental isolate fits within the broader gene content diversity observed in the analyzed dataset.

## 1. Introduction

*Staphylococcus aureus* is a globally distributed Gram-positive bacterium that occupies a central position at the interface between commensal colonization and pathogenicity, affecting humans, domestic animals, wildlife, and environmental compartments. It is consistently recognized as one of the most prevalent agents associated with community- and hospital-acquired infections and represents a major contributor to morbidity, mortality, and healthcare costs worldwide. Within the One Health framework, understood as an integrated approach that recognizes the interdependence of human, animal, and environmental health, *S. aureus* has been increasingly regarded as a paradigmatic organism due to its remarkable ecological plasticity, its capacity to colonize multiple hosts, and its ability to acquire and disseminate antimicrobial resistance and virulence determinants across interconnected human, animal, and environmental interfaces [1,2].

Antimicrobial resistance (AMR) in *S. aureus*, particularly methicillin resistance, constitutes a major public health concern. Systematic reviews and surveillance studies conducted across different continents consistently report high prevalence rates of *S. aureus* and methicillin-resistant *S. aureus* (MRSA) in clinical settings, food systems, livestock, wildlife, and environmental matrices, reinforcing the concept of a dynamic and interconnected transmission cycle [3,4,5]. In Africa, meta-analytical evidence indicates that *S. aureus* is the most prevalent member of the ESKAPE group (*Enterococcus faecium*, *S. aureus*, *Klebsiella pneumoniae*, *Acinetobacter baumannii*, *Pseudomonas aeruginosa*, and Enterobacter spp.) across human, animal, environmental, and food sources, highlighting its dominance among multidrug-resistant bacteria of global concern [2]. Similar patterns have been observed in Asia, where phenotypic resistance profiles in *S. aureus* demonstrate high levels of resistance to β-lactams and other commonly used antimicrobials, with evidence that resistant strains detected in animals or food products may later emerge in clinical settings [3,6].

Beyond clinical and agricultural contexts, wildlife has emerged as an increasingly relevant yet underexplored reservoir for *S. aureus* and MRSA. Among synanthropic species, pigeons (*Columba livia*) warrant particular attention due to their close association with urban environments and their frequent presence in hospital surroundings. Molecular studies conducted in South Africa have demonstrated that wild pigeons inhabiting areas adjacent to hospitals harbor *S. aureus* strains carrying methicillin resistance genes, multidrug resistance profiles, and clinically relevant virulence determinants [7,8]. These findings provide compelling evidence that pigeon populations may act as environmental sentinels and potential vectors for the persistence and dissemination of pathogenic *S. aureus* lineages within hospital-associated ecosystems, reinforcing their epidemiological relevance under a One Health perspective. These observations also highlight the importance of incorporating wildlife-associated reservoirs into genomic surveillance frameworks. While many previous investigations have focused primarily on phenotypic resistance profiles or targeted molecular markers, genome-wide comparative analyses are essential to determine how isolates from abiotic environmental sources and wildlife-associated hosts or biological material contribute to the broader evolutionary and epidemiological landscape of the species.

Environmental selective pressures further shape the evolutionary trajectory of *S. aureus*. In addition to antibiotic exposure, non-antibiotic stressors such as heavy metals have been shown to promote the formation of multidrug-tolerant persisted cells and to accelerate the emergence of antimicrobial resistance through oxidative stress responses and stress-response regulatory mechanisms [9]. Climate-related events, including changes in temperature, humidity, flooding, and land use, can also influence the survival, persistence, and transmission of *S. aureus* across food chains and environmental reservoirs, further increasing the complexity of antimicrobial resistance dynamics beyond traditional clinical boundaries [10]. Together, these factors underscore the need for integrated surveillance strategies that explicitly incorporate environmental drivers and wildlife interfaces into AMR monitoring frameworks.

At the genomic level, the adaptive success of *S. aureus* is underpinned by a highly dynamic genome architecture composed of a conserved core genome and a large, flexible accessory genome enriched in mobile genetic elements, resistance genes, and virulence factors. Comparative genomic and phylogenomic studies across isolates derived from humans, companion animals, livestock, and wildlife consistently reveal extensive gene content variation, reflecting ongoing horizontal gene transfer, host adaptation, and lineage diversification [1,11]. Pangenome analysis has therefore emerged as a powerful framework to capture this diversity, enabling quantification of genomic plasticity, identification of isolate-specific genetic features, and inference of population structure driven by gene presence and absence rather than solely by sequence variation.

Despite the growing recognition of the One Health relevance of *S. aureus*, significant knowledge gaps remain regarding the contribution of environmental and wildlife-associated isolates, particularly those recovered from hospital-adjacent settings to the global gene pool of the species. While numerous studies have documented phenotypic resistance patterns and targeted molecular markers in wildlife and environmental samples, comprehensive genome-wide assessments integrating such isolates into species-scale pangenome frameworks remain scarce, especially in South America and Brazil [5]. This limitation constrains our ability to place local environmental isolates within broader evolutionary and epidemiological contexts. Consequently, it becomes difficult to evaluate their potential role in the maintenance and dissemination of clinically relevant lineages.

Within this context, pangenome-based comparative genomics provides an integrative approach to address these gaps by jointly examining core genome conservation, accessory genome diversity, and isolate-specific genetic content across diverse ecological sources. By leveraging gene presence and absence patterns, this approach allows for robust assessment of genomic plasticity, population structure, and ecological adaptation, aligning directly with current calls for the incorporation of genomic data into AMR surveillance under a One Health framework [1,6].

Therefore, the present study aimed to investigate the gene content architecture and pangenome dynamics of *Staphylococcus aureus* by integrating a newly sequenced environmental isolate recovered from pigeon droppings in hospital-surrounding areas with a curated collection of publicly available reference genomes. Using a standardized pangenome framework, we sought to characterize the balance between core genome conservation and accessory genome variability, assess pangenome openness and genomic plasticity, examine gene content similarity and population structure, and identify isolate-specific genetic features within a One Health perspective. The study was designed to contextualize the environmental isolate within species-level gene content diversity rather than to infer direct transmission links between environmental and clinical reservoirs.

## 2. Materials and Methods

### 2.1. Study Overview and Analytical Scope

This study characterized the gene content architecture and pangenome dynamics of *Staphylococcus aureus* by integrating an environmental isolate (S_S3) with a curated set of publicly available reference genomes. The analytical design was structured to ensure comparability across genomes and to support downstream inference of core genome stability, accessory genome diversity, and population structure driven by gene presence/absence variation. The methodological workflow encompassed recovery of isolate S_S3 from pigeon droppings collected in hospital-adjacent outdoor areas, phenotypic confirmation of *S. aureus*, incorporation of a previously generated S_S3 genome assembly followed by reannotation with Prokka v1.14.6 to harmonize gene calling, reproducible retrieval and quality triage of RefSeq genomes followed by uniform Prokka v1.14.6 annotation, pangenome inference via orthologous gene family clustering, and downstream quantitative analyses including accumulation dynamics, gene frequency spectrum characterization, Heaps’ law modeling of pangenome openness, Jaccard-based gene content similarity estimation, multivariate ordination, hierarchical clustering, heatmap-based inspection of gene presence/absence patterns, and isolate-specific gene content assessment.

The final curated dataset comprised S_S3 and 99 RefSeq-derived genomes (total *n* = 100) and was fixed through an explicit input list (gff_list_100.txt) to ensure identical genome inclusion across computational runs. Public genomes were retrieved, uniformly reannotated, and quality-triaged prior to selection of the final diversity-aware reference panel, as detailed in Section 2.7. Each figure presented in this study can be reproduced directly from the scripts and input files available in the OSF repository, including the gene presence/absence matrix, Jaccard distance matrix, and permutation-derived accumulation datasets.

### 2.2. Environmental Sampling Area and Contextual Setting

Sampling was conducted in outdoor areas surrounding hospitals equipped with intensive care units in the southern Triângulo Mineiro region (Minas Gerais, Brazil). Sampling locations were selected based on frequent pigeon activity and the presence of surfaces that accumulate droppings, including architectural ledges, sheltered external structures, and other perching associated microhabitats proximal to hospital buildings. The study exclusively involved environmental material and did not include contact with patients, healthcare staff, or direct sampling of animals. Collection was performed under aseptic precautions to minimize exogenous contamination and to preserve the recoverability of viable bacterial cells. Sampling was conducted in publicly accessible outdoor areas, and no institutional, municipal, or ethical authorization was required under local regulations because no human participants or animal handling was involved.

As an additional technical validation step, an auxiliary Prokka audit was performed on an independent sample of complete RefSeq assemblies to verify whether the standardized annotation workflow remained operational outside the final curated dataset. This audit confirmed successful generation of valid GFF3 outputs under the same computational environment used in the study.

### 2.3. Environmental Sample Collection, Handling, and Processing

Pigeon droppings (*Columba livia*) were collected using sterile instruments and transferred into sterile containers. Samples were transported under conditions compatible with microbiological recovery and processed shortly after collection. When applicable, sample condition was recorded at collection (e.g., dry versus fresh appearance) to support descriptive interpretation and to maintain traceability of the isolate origin. Prior to culture, samples were homogenized or otherwise prepared to enable consistent inoculation onto selective and differential media.

### 2.4. Isolation, Purification, and Phenotypic Identification of Staphylococcus aureus

For recovery of staphylococci, aliquots of processed material were inoculated onto selective/differential media suitable for *Staphylococcus* spp. isolation and incubated aerobically at 36–37 °C for 24–48 h. Colonies with morphology compatible with *Staphylococcus* were subcultured to obtain pure isolates. Preliminary identification relied on Gram staining and conventional biochemical screening, including catalase and coagulase testing. A single isolate confirmed as *S. aureus* by phenotypic criteria was selected for whole-genome sequencing and designated S_S3. Pure cultures were preserved under appropriate storage conditions for subsequent genomic analyses.

### 2.5. Antimicrobial Susceptibility Testing

Phenotypic antimicrobial susceptibility of S_S3 was evaluated by disk diffusion on Mueller–Hinton agar following Clinical and Laboratory Standards Institute (CLSI) guidelines. Inoculum preparation, plate incubation, and zone measurement followed standardized laboratory procedures. Results were interpreted using Clinical and Laboratory Standards Institute breakpoint criteria (CLSI M100, 33rd edition, 2023) [12] and were subsequently compared with genomic predictions of resistance determinants derived from silico analyses. Quality control procedures were applied according to routine susceptibility testing standards to ensure methodological consistency.

### 2.6. Genome Assembly and Annotation of the S_S3 Isolate

The *Staphylococcus aureus* isolate S_S3 analyzed in the present study is the same environmental isolate reported in our prior study [13] in which it was sequenced and assembled using an Illumina-based de novo approach. Here, the previously generated assembly was reused for standardized reannotation and downstream comparative pangenome analyses. The curated assembly, together with its original annotation files, was archived as an analysis bundle (S_S3-s.aureus.tar.gz).

In the present study, to ensure full comparability with the RefSeq-derived genomes and to minimize annotation-driven bias in gene presence/absence inference, the S_S3 assembly was reannotated using Prokka (v1.14.6) with the same standardized parameters applied to the public genomes (default settings unless otherwise specified). The resulting Prokka-generated GFF3 file was used as the standardized input for downstream pangenome reconstruction. The assembled genome of *Staphylococcus aureus* isolate S_S3 was deposited in the NCBI GenBank repository under BioProject accession PRJNA1415265 and BioSample accession SAMN54927991. The GenBank whole-genome shotgun accession number will be provided upon public release of the record.

### 2.7. Public Genome Retrieval and Dataset Construction

To place the environmental isolate S_S3 within the broader genomic diversity of the species, we initially retrieved a large collection of *Staphylococcus aureus* genomes from the NCBI RefSeq repository using an automated download workflow. This initial retrieval yielded 3780 genome FASTA files available under the applied query constraints at the time of access. From this collection, 1000 genomes were selected by simple random sampling without replacement using a scripted procedure with a fixed seed, thereby ensuring exact reproducibility of the candidate set used for standardized reannotation.

All genomes in this candidate subset were processed with Prokka v1.14.6 under identical parameters to generate standardized GFF3 annotations suitable for downstream pangenome reconstruction. Among these 1000 genomes, 323 yielded valid Prokka GFF3 files and were therefore eligible for direct inclusion in Roary. The remaining 677 genomes did not generate standardized GFF3 outputs suitable for downstream processing and were excluded at this stage. Because gene presence and absence inference is highly sensitive to annotation heterogeneity, only genomes that successfully passed the same standardized annotation workflow were considered for further analysis.

From the 323 successfully reannotated genomes, the final 99 RefSeq genomes were selected through a reproducible curation step designed to retain high quality assemblies while reducing overrepresentation of near identical genomes. This curation considered assembly contiguity metrics, including contig count and N50, genome completeness and contamination estimates based on CheckM2 and BUSCO, metadata consistency, including stable RefSeq accessions and traceable assembly records, and pairwise gene content similarity inspection to reduce redundancy among genomes with highly overlapping repertoires. The purpose of this step was not to generate an exhaustive representation of all publicly available *S. aureus* diversity, but rather to construct a diversity-aware, reproducible, and computationally tractable reference panel for standardized pangenome inference.

These 99 reference genomes were combined with the environmental isolate S_S3 to form the final dataset of 100 genomes. To prevent annotation-related bias in gene presence and absence inference, all genomes in the final dataset were uniformly annotated under the same Prokka v1.14.6 configuration. The final dataset composition was frozen through an explicit input list of GFF3 files, which served as the direct input for all Roary executions and ensured identical genome inclusion across computational runs.

To improve transparency and reproducibility, the OSF repository provides the accession list from the initial RefSeq retrieval, the randomly sampled 1000 genome candidates set, the subset yielding valid Prokka GFF3 outputs, the frozen final genome inclusion list, and the scripts used for each processing step. NCBI genome data were accessed on 3 January 2026. All genomes included in the final dataset were further subjected to standardized quality assessment, as detailed in Section 2.9.

### 2.8. MLST and Clonal Complex Assignment

Multilocus sequence typing (MLST) profiles were determined for all genomes included in the final dataset (*n* = 100) using the PubMLST *Staphylococcus aureus* scheme. Sequence types (STs) were assigned based on allelic profiles derived from assembled genomes. Clonal complexes (CCs) were inferred from ST assignments using eBURST-based definitions implemented in the PubMLST database. For sequence types without a defined clonal complex, genomes were classified as “unassigned CC”. All ST and CC annotations used in this study are provided in Supplementary Table S2.

### 2.9. Genome Quality Assessment and Filtering Criteria

To ensure the inclusion of high-quality genomes and to minimize bias in pangenome inference, all genomes retained in the final dataset were subjected to standardized quality assessment before downstream analyses. Genome completeness and contamination were estimated using CheckM2, a machine learning-based framework for genome quality evaluation. In parallel, genome completeness was independently assessed with BUSCO using the bacteria lineage dataset. Assembly contiguity was summarized using total assembly size, number of contigs, and N50 values calculated from genome FASTA files.

Genomes were retained for the final dataset if they met the following quality criteria: high completeness, typically at least 95 percent based on CheckM2 and or BUSCO, low contamination, typically at most 5 percent based on CheckM2, and acceptable assembly contiguity based on inspection of contig count and N50 values. These thresholds are consistent with commonly applied standards in bacterial comparative genomics and pangenome studies.

In the final dataset, CheckM2 completeness was 100 percent for all genomes, CheckM2 contamination remained below 2 percent in all cases, and BUSCO completeness remained high, ranging from 97.4 percent to 100 percent. A complete summary of assembly statistics and quality metrics for all genomes included in the study is provided in Supplementary Table S1, including genome size, GC content, number of contigs, N50, CheckM2 completeness and contamination estimates, and BUSCO completeness scores. These results support the suitability of the final genome panel for comparative pangenome analysis.

### 2.10. Pangenome Reconstruction and Orthologous Gene Family Clustering

Pangenome inference was performed using Roary (v3.13.0), which clusters orthologous genes across genomes based on Prokka-standardized GFF3 annotations. Roary was executed with parameters enabling paralog handling and strict core alignment (-e -n), multithreading (-p 4), and default clustering settings for sequence identity and core definition (identity threshold = 95% and core threshold = 99%). All genomes were provided through a fixed and auditable input file list comprising S_S3 and 99 RefSeq-derived reference genomes. This frozen file list ensured identical dataset composition across computational runs and full reproducibility of pangenome inference. Primary outputs included the non-redundant gene family catalog and the gene presence–absence matrix, which served as the basis for all downstream quantitative analyses. The final inferred pangenome comprised 8366 non-redundant gene families across the 100 analyzed genomes.

### 2.11. Definition of Core, Strict Core, Accessory, Shell, and Cloud Gene Fractions

Gene family prevalence was defined by the number of genomes in which each gene family was detected. Core genes were defined under the conventional Roary threshold as gene families present in 99–100% of genomes. Within this category, strict core genes represent the subset detected in 100% of genomes and were analyzed separately to distinguish universally conserved loci from genes that are nearly universal but absent in a small number of genomes. Accessory genes comprised all gene families not belonging to the Roary-defined core. When required for interpretability, accessory genes were further partitioned into intermediate frequency shell genes and rare cloud genes according to prevalence-based cutoffs used in standard pangenome reporting. These prevalence definitions were applied consistently across accumulation analyses, frequency spectrum visualizations, and heatmap-based inspections. For heatmap visualization, the shell fraction was operationally defined as gene families with intermediate prevalence (presence frequency 0.15–0.85 across genomes), consistent with the filtering strategy described in Section 2.16.

### 2.12. Pangenome and Core Genome Accumulation Analysis

**Figure 1:**
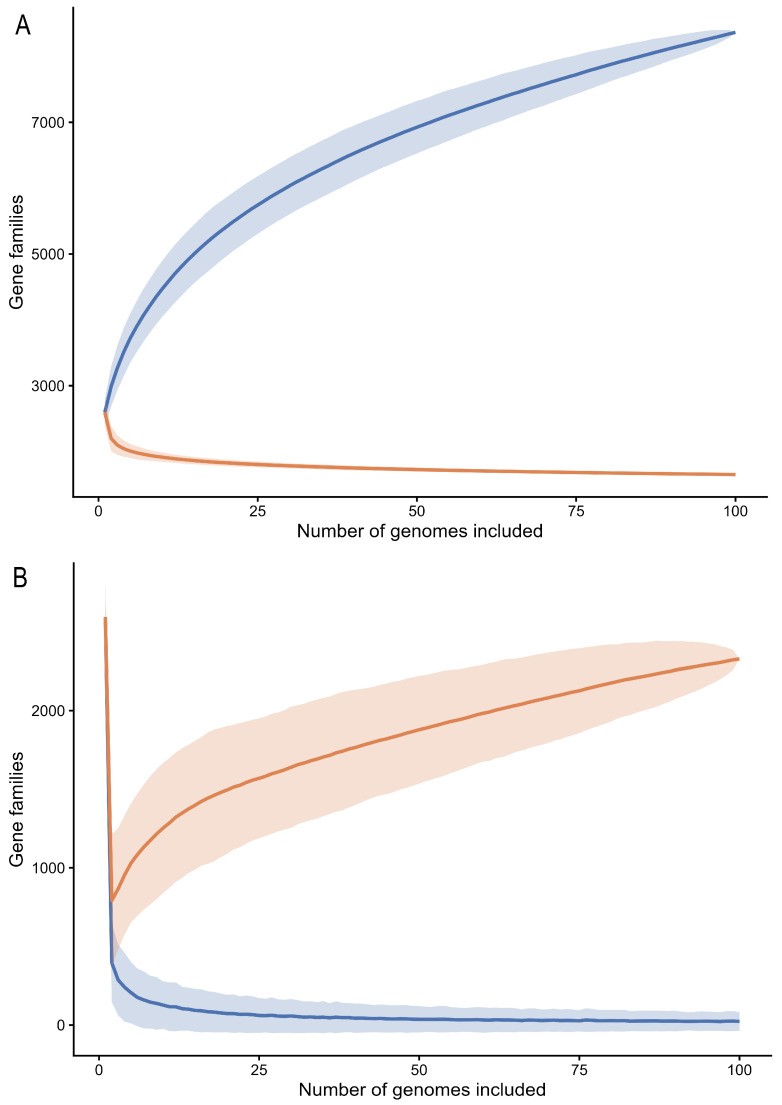
Pangenome structure, conservation, and gene acquisition dynamics in *Staphylococcus aureus*. (**A**) Pangenome and core genome accumulation curves derived from 100 genomes, including the newly sequenced isolate S_S3. The *x*-axis represents the number of genomes sequentially incorporated into the analysis, and the *y*-axis indicates the number of gene families. The pangenome curve (blue) shows the progressive increase in non-redundant gene families, while the core genome curve (orange) represents the strictly conserved core genome (genes present in 100% of genomes). Shaded areas indicate the 95% confidence intervals derived from 1000 random permutations of genome inclusion order. (**B**) Dynamics of novel and unique gene acquisition during pangenome construction. The blue curve indicates the number of novel gene families introduced by each additional genome, whereas the orange curve shows the cumulative number of unique genes (gene families present in only one genome) observed during sequential genome incorporation. The trajectories illustrate continued gene discovery, pronounced inter-genome variability in gene contribution, and the persistent emergence of strain-specific genes across the dataset.

Accumulation dynamics were assessed by sequentially incorporating genomes into the pangenome construction and recording the cumulative number of distinct gene families (pangenome) and the number of conserved families meeting the core definition (core genome). To mitigate the influence of genome addition order, 1000 random permutations of genome inclusion were generated, and accumulation curves were summarized as mean trajectories across permutations. A fixed random seed (seed = 1234) was applied to ensure exact reproducibility of permutation ordering. The curves shown in Figure 1 represent the mean accumulation trajectories calculated across the 1000 random permutations of genome inclusion order. This approach enabled assessment of convergence behavior for both pangenome size and core genome stabilization and supported inference of sustained gene discovery at increasing sampling depth. In parallel, the number of newly introduced gene families per genome addition was computed to quantify heterogeneity in gene contribution among isolates. Unique gene accumulation was calculated as the cumulative number of gene families present in only a single genome across the dataset.

### 2.13. Heaps’ Law Modeling of Pangenome Openness

Pangenome openness was quantified using Heaps’ law modeling applied to the cumulative pangenome growth curve. The model was defined as P(n) = k × n^γ where P(n) represents the total number of gene families observed after incorporation of *n* genomes, k is a proportionality constant, and γ is the scaling exponent describing pangenome openness. Model fitting was performed on the mean pangenome accumulation trajectory obtained from 1000 random genome order permutations (Section 2.10), thereby minimizing ordering effects on parameter estimation. Parameters k and γ were estimated by nonlinear least squares regression. Under the standard interpretation for microbial pangenomes, γ values below 1 indicate an open pangenome in which newly sequenced genomes continue to contribute novel gene families as sampling increases. Parameter estimates were reported and interpreted in conjunction with empirical accumulation curves to support conclusions regarding ongoing gene acquisition and genomic plasticity within the dataset.

### 2.14. Gene Frequency Spectrum Analysis

The gene frequency spectrum was computed from the Roary gene presence/absence matrix by tallying the number of gene families observed at each prevalence level from singletons to universally present genes. The resulting histogram was used to characterize the dominance of low-frequency gene families and to contrast rare accessory content with the compact conserved backbone. The strict core subset was explicitly reported as the frequency class representing gene families present in all genomes, and it was distinguished from the broader Roary-defined core fraction to maintain conceptual clarity. Frequency distributions were used to support interpretation of cloud and shell contributions to overall pangenome structure.

### 2.15. Gene Content Similarity Computation Using Jaccard Distance

Genome-wide similarity in gene content was quantified using the Jaccard index computed from the binary presence/absence profiles of gene families. Pairwise Jaccard distances were computed for all genome pairs to generate a genome by genome, dissimilarity matrix. This matrix was used both for visualization of global similarity patterns and as the basis for multivariate and hierarchical analyses of population structure. Jaccard-based dissimilarity was selected because it is well suited to binary gene content data and emphasizes shared versus differential accessory repertoires while remaining insensitive to joint absences. To assess dataset representativeness and potential redundancy arising from the random genome selection strategy, summary statistics and the full distribution of pairwise Jaccard distances were evaluated, including mean, range, and the proportion of highly similar genome pairs. This analysis was used as a post hoc quantitative assessment of diversity coverage across the dataset.

### 2.16. Multivariate Ordination by Principal Component Analysis

To explore low dimensional structure in gene content variation, principal component analysis (PCA) was performed directly on the binary gene presence/absence matrix generated by Roary. The first two principal components were used for visualization and interpretation of clustering behavior, and the proportion of variance explained by each component was reported. The newly sequenced isolate S_S3 was highlighted in ordination space to assess whether its gene content profile aligned with major clusters or exhibited outlier behavior relative to the reference collection. PCA was performed in Python 3.13 using scikit-learn version 1.7.2 (sklearn.decomposition.PCA), which mean-centers features by default prior to decomposition.

### 2.17. Hierarchical Clustering and Dendrogram Reconstruction

Hierarchical clustering was performed using the pairwise Jaccard distance matrix computed from gene presence/absence profiles. Clustering was conducted using average linkage agglomeration to generate a dendrogram representing gene content relatedness among genomes. Branch heights were interpreted as increasing gene content dissimilarity. The placement of S_S3 within the dendrogram was used to contextualize its relationship to subsets of reference genomes and to support integration of ordination and clustering results.

### 2.18. Heatmap Generation for Accessory and Shell Gene Distributions

Binary presence/absence heatmaps were constructed to visualize gene level heterogeneity across genomes and to resolve structured variation within the accessory genome. Two heatmaps were generated. First, an accessory gene heatmap was built using all non-core gene families derived from the Roary presence/absence matrix (i.e., excluding gene families meeting the core definition under the applied threshold). Second, a shell gene heatmap was generated by restricting the analysis to gene families with intermediate prevalence across the dataset (presence frequency between 0.15 and 0.85), thereby emphasizing loci contributing most strongly to between cluster differentiation.

When the number of eligible shell gene families was large, the subset of genes with the highest variance in presence/absence across genomes (up to 300 gene families) was retained to improve visualization readability while preserving discriminatory signal. Rows corresponded to gene families and columns to genomes. Genome ordering followed the hierarchical clustering-derived dendrogram based on pairwise Jaccard distances, facilitating visualization of modular gene blocks and patterns of genes occurring together. Presence and absence states were color coded, and visual boundaries applied after genome ordering were used to delineate contrasts in gene composition between genome subsets. Heatmaps were generated using Python-based visualization libraries (matplotlib and seaborn).

### 2.19. Isolate-Specific Gene Identification and Functional Inspection in S_S3

To quantify isolate level novelty, gene families were classified as S_S3-specific if they were present exclusively in S_S3 and absent from all other genomes in the dataset. In addition, S_S3 was evaluated for missing core genes by intersecting its gene family profile with the Roary-defined core set. For S_S3-specific families, annotation outputs were inspected to summarize functional categories, including the proportion of hypothetical proteins and the presence of functionally annotated loci. Functionally notable isolated exclusive genes, were extracted based on annotation and resistance-associated signatures, supporting interpretation of biologically relevant accessory contribution while maintaining consistency, with the frequency-based framework used across the dataset. To refine the functional characterization of isolate-specific genes, all S_S3-specific gene families were subjected to homology searches using DIAMOND against the UniProt Swiss-Prot database. The best hits were retained, and functional assignments were compared against the initial Prokka annotation to identify conserved domains or putative functions not previously detected.

### 2.20. Software, Computational Environment, and Reproducibility Practices

All analyses were performed on Ubuntu 22.04.3 LTS running under Windows Subsystem for Linux 2 (WSL2; kernel 6.6.87.2-microsoft-standard-WSL2) using Conda-managed environments. Pangenome inference and downstream analyses were executed in a dedicated Conda environment containing Roary and its dependencies.

To ensure full computational traceability, software versions, system metadata, and dependency information were automatically recorded at runtime and stored in a VERSIONS.txt file generated by the execution pipeline.

Workflow execution was orchestrated through scripted pipelines implementing explicit input validation, standardized file naming, frozen genome inclusion lists, and automated export of all primary Roary outputs and derived analysis tables. This design guarantees exact reproduction of all reported results upon rerunning of the provided scripts.

### 2.21. Data Availability

All datasets and computational resources supporting the findings of this study are publicly available. Derived datasets, including gene family presence and absence outputs, pangenome accumulation series, gene frequency spectrum tables, Jaccard distance matrices, and clustering and ordination results, are provided as Supplementary Materials and deposited in the associated Open Science Framework repository.

The OSF repository also includes the accession lists for the initial RefSeq retrieval, the randomly sampled candidate set, the successfully reannotated subset, the frozen final genome inclusion list, quality control summary tables, and the scripts required to reproduce genome retrieval, annotation, dataset curation, pangenome inference, and downstream analyses.

All scripts required to reproduce genome retrieval, uniform annotation, pangenome inference, and downstream analyses, including genome download workflows, Prokka annotation pipelines, Roary execution scripts, frozen genome inclusion lists, and environment specifications, are available in the registered OSF project. The complete reproducibility repository is accessible at the Open Science Framework under registration https://doi.org/10.17605/OSF.IO/3C4B9.

## 3. Results

### 3.1. Dynamics of Pangenome Expansion, Core Genome Conservation, and Genomic Plasticity in Staphylococcus aureus

Using a uniformly annotated dataset comprising 100 *Staphylococcus aureus* genomes, including the environmental isolate S_S3 (previously sequenced and assembled and reannotated here with Prokka v1.14.6 for harmonization) and 99 publicly available RefSeq genomes, we characterized the structure, accumulation behavior, and plasticity of the species’ global gene repertoire. The Roary gene presence/absence matrix identified 8366 non-redundant gene families, revealing a large and heterogeneous pangenome across the collection.

Analysis of genome accumulation dynamics demonstrated a sustained expansion of the pangenome as additional genomes were incorporated (Figure 1A). Genomes were sequentially added along the *x*-axis from 1 to 100, ensuring a strictly increasing sampling order. To minimize biases associated with genome inclusion order, accumulation trajectories were calculated as the mean values derived from 1000 random permutations of genome addition. Under this framework, the total number of gene families increased progressively, reaching approximately 8.36 k gene families at the end of the sequential sampling. Across permutations, the terminal pangenome size remained highly consistent, converging within a narrow range (~8360–8366 gene families). This convergence supports the robustness and reproducibility of the inferred pangenome size and indicates that the observed diversity is not an artifact of genome ordering.

In contrast to the expanding pangenome, the conserved gene fraction exhibited a decreasing trend during genome incorporation, followed by stabilization as sampling depth increased (Figure 1A). Under a strict definition of core genes (gene families present in 100% of genomes), the core genome converged to approximately 1651 gene families, reflecting a relatively stable genomic backbone shared across all isolates. This stability contrasts with the progressive expansion of the accessory gene pool observed as additional genomes were incorporated into the analysis, highlighting the coexistence of a conserved genomic core and a highly dynamic accessory repertoire in *Staphylococcus aureus*.

To further assess pangenome openness and genomic plasticity, we examined the dynamics of novel and unique gene acquisition during sequential genome addition (Figure 1B). These trajectories were computed using the same permutation framework described above, ensuring that genome inclusion order did not influence the overall patterns observed. The number of newly introduced gene families contributed by each genome varied markedly, ranging from only a few genes to several hundred per genome. This heterogeneity indicates substantial differences among isolates in their contribution to the overall gene repertoire, reflecting uneven distributions of accessory genetic material within the population.

Despite this variability, the overall trend shows that additional genomes continue to introduce previously unobserved gene families during the accumulation process (Figure 1B). As genome sampling increases, the number of newly identified genes per genome gradually decreases. However, novel gene families continue to appear even at later stages of sampling, indicating that the pangenome remains open. Additional genomes still contribute to hundreds of previously unobserved genes, although at a lower rate than in the early stages of sampling.

In parallel, the accumulation of unique genes, defined as gene families present in only a single genome, revealed a steady enrichment of strain-specific content across the dataset (Figure 1B). The cumulative number of unique gene families (singletons) across the dataset, computed during sequential genome incorporation, increased continuously and reached approximately 2328 gene families when accumulated across the full dataset. This cumulative metric reflects the progressive discovery of strain-restricted gene families as additional genomes are sampled, whereas the final frequency spectrum captures the number of singletons present at the end of dataset assembly (Section 3.2). Together, these results indicate that a substantial fraction of the *S. aureus* gene pool is composed of rare or isolate-specific elements that are introduced sporadically rather than uniformly.

Taken together, the concordant trajectories of pangenome expansion, core genome stabilization, and sustained acquisition of novel and unique genes demonstrate pronounced genomic flexibility in *Staphylococcus aureus*. A relatively stable core genome is continuously complemented by a large and dynamic accessory gene reservoir, likely shaped by horizontal gene transfer, mobile genetic elements, and niche-specific selective pressures. These results collectively highlight ongoing gene content diversification in *S. aureus* within a dataset of 100 genomes. Raw accumulation series and gene frequency outputs are provided as Supplementary Data in the associated repository to ensure full reproducibility.

### 3.2. Gene Frequency Spectrum Highlights a Dominant Accessory Genome and a Compact Conserved Backbone

**Figure 2:**
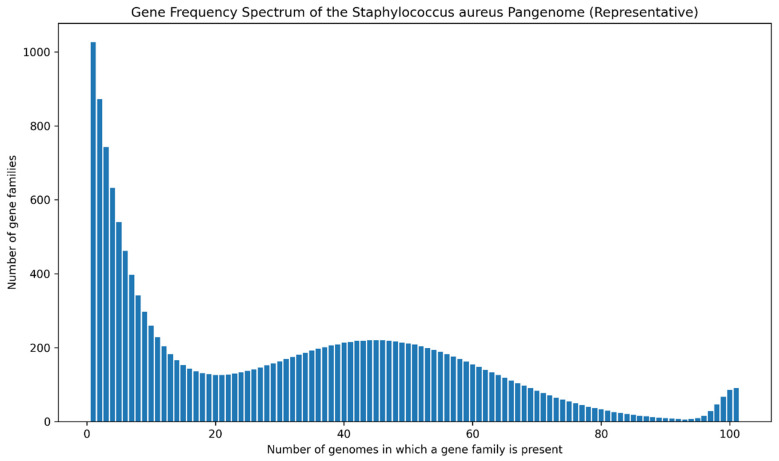
Gene frequency spectrum of the *Staphylococcus aureus* pangenome. The histogram shows the distribution of gene families according to the number of genomes in which each family is detected across the 100 analyzed genomes. The spectrum is strongly skewed toward low-frequency genes, with a large proportion of gene families occurring in only one or a few genomes (cloud genome), reflecting extensive accessory gene diversity. A smaller fraction of genes displays intermediate frequencies (shell genome), forming a gradual continuum rather than discrete frequency classes. At the extreme right of the spectrum, a limited subset of strictly conserved, non-redundant gene families are detected in all analyzed genomes, representing conserved loci as defined by the Roary clustering pipeline after its standard paralog handling step. Together, this distribution illustrates a classical open pangenome architecture characterized by a compact conserved backbone overlaid by a large and dynamic accessory gene pool.

The gene frequency spectrum across the *Staphylococcus aureus* pangenome reveals a highly uneven distribution of gene presence among strains, reflecting a pronounced separation between conserved and accessory genomic components (Figure 2). Analysis of gene occurrence across the 100 genomes demonstrates that the pangenome is overwhelmingly dominated by low-frequency gene families, while only a limited subset of loci is consistently detected across most or all genomes, a hallmark of an open and highly plastic genomic architecture.

A striking feature of the frequency distribution is the strong predominance of rare genes. A total of 1031 gene families were detected in only a single genome, representing the largest individual frequency class and defining a substantial cloud genome. In addition, several hundred gene families were present in fewer than five genomes, collectively accounting for a major proportion of the accessory gene pool (Figure 2). This abundance of low-frequency genes is indicative of continuous gene turnover, likely driven by horizontal gene transfer, mobile genetic elements, and niche or lineage-specific selective pressures.

At the opposite extreme of the frequency spectrum, a much smaller subset of gene families is detected across all analyzed genomes. These genes correspond to strictly conserved, unique loci that remain invariant after paralog resolution and represent the most stable functional backbone of the species. Importantly, this subset does not encompass the full core genome as defined by the Roary-based presence threshold (99–100% of genomes), which was quantified separately in the pangenome accumulation analysis (Figure 1). Instead, these strictly conserved gene families highlight a reduced set of essential functions under strong purifying selection.

Between these two extremes lies a comparatively modest shell genome, composed of gene families present in an intermediate number of strains (approximately 15–95 genomes). This component displays a gradual decline in gene counts with increasing frequency, suggesting a continuum of gene retention rather than sharply defined boundaries between pangenome compartments. Shell genes are likely to contribute to lineage-specific traits, ecological specialization, and adaptive flexibility across different host-associated and environmental contexts.

Collectively, the gene frequency spectrum observed here is characteristic of a classical open pangenome, in which a stable and conserved genomic backbone is overlaid by a vast, dynamic accessory gene reservoir. This architecture reinforces the view that *S. aureus* maintains evolutionary flexibility through persistent remodeling of its accessory genome, while preserving a compact set of essential functions.

### 3.3. Accessory Gene Content Structures the Population into Distinct Genomic Clusters

**Figure 3:**
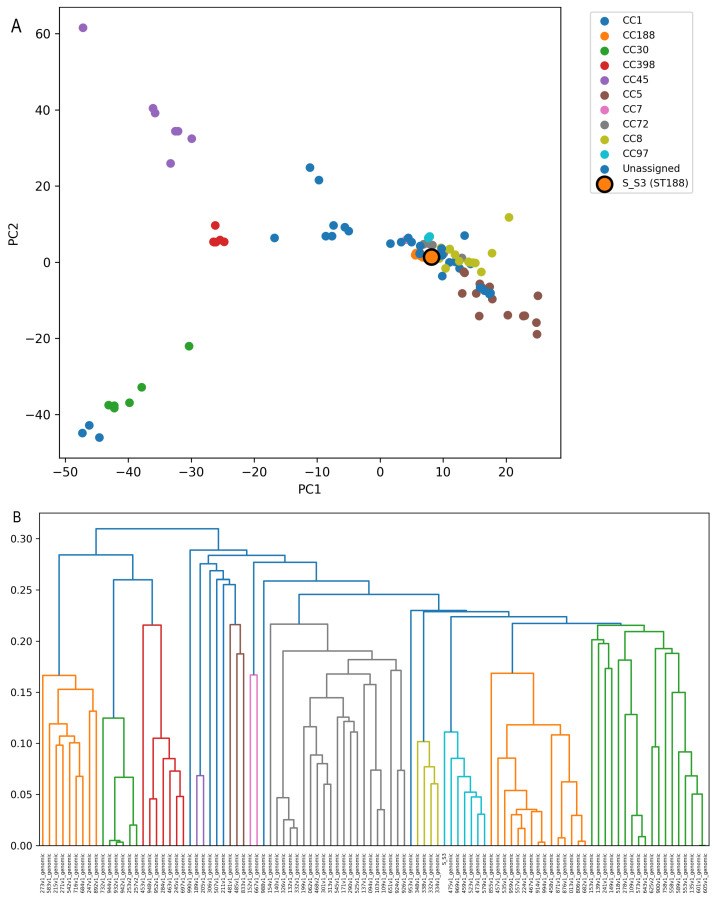
Accessory genome-driven population structure of *Staphylococcus aureus*. (**A**) Principal component analysis (PCA) of genomes based on the Roary gene presence/absence matrix with MLST clonal complex (CC) annotation. Each point represents one genome and is colored according to its assigned clonal complex (CC). The environmental isolate S_S3 (ST188, CC188) is highlighted with a black-edged orange marker. Axes represent the first two principal components (PC1 and PC2), with the percentage of variance explained indicated in parentheses. The clustering pattern shows strong correspondence between accessory genome variation and established MLST-based population structure. (**B**) Hierarchical clustering dendrogram constructed from pairwise Jaccard distances computed on gene presence/absence profiles among the analyzed genomes. The *y*-axis represents Jaccard distance, where higher values indicate greater dissimilarity in gene content. The position of S_S3 within the dendrogram highlights its gene content relationships to reference genomes and its placement within the overall population structure.

To explore how gene content variation organizes the *Staphylococcus aureus* population, we examined multivariate and hierarchical patterns derived from the Roary presence/absence matrix using PCA (on the binary matrix) and Jaccard-based dissimilarity for clustering (Figure 3A,B). Across the 100 uniformly annotated genomes, per-genome gene counts were broadly comparable yet variable, ranging from 2411 to 2789 genes per genome, with the newly sequenced isolate S_S3 carrying 2582 genes (per-genome statistics table). This dispersion indicates that, even within a relatively constrained genome size window, differences in accessory gene complement are sufficient to drive measurable population structure.

A principal component analysis (PCA) performed directly on the binary gene presence/absence matrix revealed clear separation of genomes into multiple clusters in low-dimensional space (Figure 3A). Although the first two components captured a modest fraction of total variance (PC1 = 14.1% and PC2 = 8.8%), the clustering pattern was pronounced, consistent with discrete gene content configurations across lineages. Notably, S_S3 mapped within one of the major clusters rather than appearing as an outlier, supporting its placement within an established genomic background defined by shared accessory content (Figure 3A).

To explicitly link clustering driven by the accessory genome with established population structure, genomes were annotated with MLST sequence types and clonal complexes (CCs), and these labels were overlaid onto the PCA ordination (Figure 3A; Supplementary Table S2). This annotation revealed that the observed clustering patterns are strongly associated with known *S. aureus* clonal complexes. Distinct clusters correspond predominantly to CC-defined lineages, including CC1, CC5, CC8, CC30, CC45, and CC398, indicating that accessory genome variation is structured in a lineage-dependent manner.

Genomes belonging to the same CC consistently grouped together in PCA space, demonstrating that accessory gene content recapitulates the established MLST-based population structure. This concordance indicates that gene presence/absence variation captures biologically meaningful lineage relationships beyond core genome phylogeny. The environmental isolate S_S3 (ST188, CC188) clustered within the CC188-associated group and did not appear as an outlier, further supporting its integration within a recognized *S. aureus* lineage. This placement reinforces that S_S3 shares a common accessory genome background with other members of its clonal complex, consistent with its limited number of isolate-specific genes.

Hierarchical clustering of genomes based on pairwise Jaccard distances corroborated the PCA-defined structure and provided an explicit topology of relatedness (Figure 3B). The dendrogram resolved several coherent clades separated by moderate-to-high branch heights, reflecting increasing dissimilarity in gene content among groups. Pairwise Jaccard distances spanned a broad range (0.00–0.39; mean = 0.26), indicating moderate-to-high variation in gene content across genomes. In this context, the positioning of S_S3 within a defined branch of the dendrogram further supports its close affinity to a subset of genomes with more similar gene repertoires, while still maintaining measurable distances to more divergent groups (Figure 3B).

**Figure 4:**
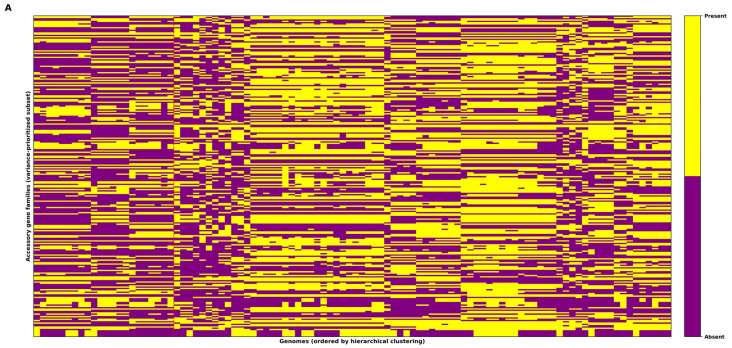

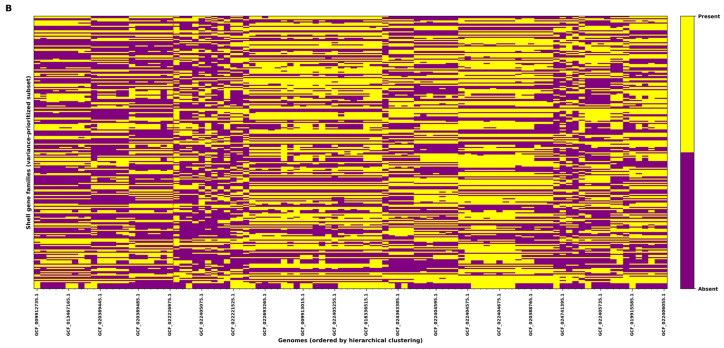
Presence–absence heatmaps of accessory and shell genes in the *Staphylococcus aureus* pangenome. (**A**) Heatmap showing the distribution of accessory (non-core) gene families across the 100 analyzed genomes, based on a binary presence (1)/absence (0) matrix. Rows correspond to gene families and columns to individual genomes. The mosaic pattern highlights structured heterogeneity, with blocks of shared gene presence across subsets of genomes consistent with lineage-associated gene content modules. (**B**) Heatmap restricted to shell genes, defined here as gene families with intermediate prevalence across the dataset (presence frequency 0.15–0.85). Shell genes exhibit more structured, block-like presence patterns spanning multiple genomes, indicating no random distribution, and reinforcing population stratification by accessory content. When necessary for readability, gene families were prioritized by highest presence/absence variance (up to 300 loci). Genome ordering follows hierarchical clustering based on pairwise Jaccard distances. The vertical dashed red line marks a visual boundary between genome subsets displaying contrasting shell gene composition after genome ordering. Color scale represents gene presence (yellow) and absence (purple).

Collectively, these results indicate that *S. aureus* genomes segregate into structured gene content clusters, with both ordination and hierarchical approaches converging on the same conclusion: population organization in this dataset is strongly shaped by accessory gene composition, as captured by Jaccard-based dissimilarity (Figure 3A,B). The strong correspondence between clustering patterns and MLST-defined clonal complexes further demonstrates that accessory genome variation is not randomly distributed but instead reflects underlying lineage structure. Together, Figure 3A,B provide a global genome-level view of gene content organization; in the next section, we complement this overview with gene-level presence/absence heatmaps (Figure 4) that resolve the specific accessory and shell loci underlying these clusters.

### 3.4. Accessory and Shell Gene Heatmaps Reveal Structured Heterogeneity in the S. aureus Pangenome

The presence/absence heatmaps provide a high resolution representation of accessory gene distribution across the 100 *Staphylococcus aureus* genomes, revealing pronounced heterogeneity and clear structure in gene content variation (Figure 4A,B). In the accessory gene heatmap (Figure 4A), the binary matrix displays a strong mosaic pattern, in which large, contiguous blocks of shared gene presence across subsets of genomes coexist with extensive regions of absence. This configuration indicates that accessory gene repertoires are not uniformly dispersed across the dataset; instead, they are organized into modular gene sets that tend to occur within specific genomic backgrounds, consistent with lineage-associated gene content. Where necessary, heatmaps were rendered using a variance-prioritized subset of loci for readability (Section 2.16).

This visual organization is supported by the underlying frequency-based pangenome partitioning. A strict core of 1651 gene families was detected in all 100 genomes (frequency = 1.0), representing the conserved core functional backbone. The simultaneous presence of a compact, universally conserved core alongside a vast reservoir of rare, strain-restricted genes provide a mechanistic explanation for the contrast seen in Figure 4A, where localized “islands” of presence emerge against widespread absence across genomes.

When the analysis is restricted to the shell fraction, the resulting heatmap (Figure 4B) reveals a more structured pattern than the broader accessory matrix. Rather than being dominated by singletons, shell genes, defined here as gene families with intermediate prevalence across genomes (presence frequency 0.15–0.85), tend to form coherent presence blocks spanning multiple strains. These blocks suggest that shell genes contribute disproportionately to group differentiation, capturing genomic signatures shared within subsets of related isolates while remaining absent from others. The block-like organization observed in Figure 4B indicates that intermediate frequency genes are not randomly distributed, but instead align with population structure, plausibly reflecting stable, lineage-associated traits that may be selectively maintained under ecological or host-associated conditions.

Taken together, Figure 4A,B show that the *S. aureus* pangenome is characterized by a conserved core present across all genomes and a highly variable accessory reservoir enriched for rare genes, while the shell component provides a structured intermediate layer that reinforces genomic stratification across the dataset.

### 3.5. Genomic Composition and Isolate-Specific Gene Content of the S_S3 Genome

**Figure 5:**
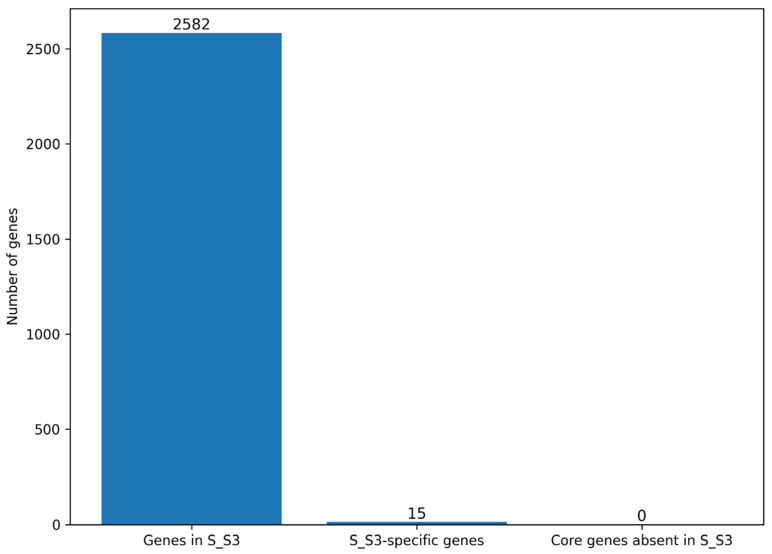
Genomic composition and isolate-specific gene content of the *Staphylococcus aureus* S_S3 isolate. The bar chart summarizes the gene content of the newly sequenced isolate S_S3 in the context of the *S. aureus* pangenome inferred from 100 genomes. The total number of genes annotated in S_S3 (2582 genes) is shown alongside the number of isolate-specific gene families (15 genes) and the number of core genes absent from this genome (0 genes). Isolate-specific genes were defined as gene families present exclusively in S_S3 across the dataset, whereas core genes correspond to gene families detected in 99–100% of all analyzed genomes. The absence of missing core genes indicates complete retention of the conserved genomic backbone, while the limited number of unique genes (~0.18% of the total pangenome) highlights a modest yet biologically relevant contribution of accessory genetic material. Together, these features indicate strong conservation of the core genome together with low-frequency, and isolate-specific variation within the *S. aureus* population.

To contextualize the genomic contribution of the newly sequenced isolate S_S3 within the *Staphylococcus aureus* pangenome, we performed a targeted assessment of its gene content relative to the global gene repertoire inferred from the 100-genome dataset. As summarized in Figure 5, the S_S3 genome comprises a total of 2582 annotated genes, all of which were assigned to gene families in the dataset-wide pangenome. Within this dataset, 15 gene families were exclusive to S_S3, reflecting a limited but distinct isolate-specific accessory contribution. Notably, no core genes were absent from S_S3, indicating full retention of the conserved genomic backbone defined for the species under the applied core gene threshold.

Despite complete representation of the core genome, S_S3 contributes a small number of isolate-specific elements to the overall pangenome. These 15 unique gene families correspond to approximately 0.18% of the full pangenome (15 of 8366 gene families). It is important to note that this classification reflects uniqueness relative to the selected genome collection and may be influenced by dataset composition and sampling breadth. Therefore, these genes should be interpreted as dataset-specific rather than universally exclusive to S_S3.

Nevertheless, the low fraction of unique genes is consistent with the overall pattern of extensive core genome conservation observed across the dataset, while still reflecting the presence of rare and strain-specific accessory genetic elements. The unique gene set of S_S3 is predominantly composed of hypothetical proteins, several of which are distributed across accessory genomic regions, suggesting possible acquisition through horizontal gene transfer or lineage-associated genomic rearrangements.

To refine the functional interpretation of isolate-specific genes beyond the initial Prokka-based annotation, all S_S3-specific gene families were subjected to homology searches against the UniProt Swiss-Prot database using DIAMOND. This analysis identified significant matches for a subset of genes, revealing putative functional assignments including transporters, metal uptake systems, resistance-related proteins, and mobile genetic elements such as transposases. Notably, genes with high-confidence hits included homologs of nickel transporters, bacitracin resistance-associated proteins, and membrane-associated transport systems, suggesting plausible roles in environmental persistence or stress response, although these inferences remain annotation-based and require experimental validation. In contrast, several genes remained without significant matches, indicating a fraction of uncharacterized or highly divergent sequences.

Overall, these results indicate that while a subset of isolate-specific genes encodes proteins with plausible biological functions, the majority remain poorly characterized, consistent with the expected enrichment of hypothetical proteins within the low-frequency accessory genome. These findings reinforce that accessory genome variability includes both functionally informative elements and a reservoir of poorly annotated sequences, highlighting current limitations in functional annotation databases rather than a lack of biological relevance.

Among the isolate-specific genes, one functionally annotated gene encodes a lincosamide resistance protein, indicating the presence of a resistance-associated determinant unique to S_S3 within the analyzed dataset. Importantly, this classification reflects exclusivity relative to the 100-genome panel and does not imply rarity in the broader *Staphylococcus aureus* population. In addition, the gene efeB, encoding a deferrochelatase/peroxidase-related protein involved in iron metabolism and oxidative stress response, was also detected exclusively in S_S3 within this dataset, which may be consistent with stress-related or niche-associated functions, although no functional validation was performed in this study.

The genomic distribution of these unique genes reveals their localization within discrete accessory regions rather than within the conserved genomic backbone, reinforcing their classification as low-frequency or rare elements. Gene length among S_S3-specific families ranged from approximately 150 to more than 560 nucleotides, indicating structural heterogeneity rather than systematic truncation or annotation artifacts. Standard annotation and quality-control procedures did not classify these genes as low-confidence predictions, supporting their validity as genuine genomic features.

Consistent with this observation, the genomic profile of S_S3 reveals strong conservation of the core genome together with a modest but biologically relevant contribution from accessory genes (Figure 5). The absence of missing core genes, combined with the limited yet functionally diverse set of unique genes, positions S_S3 as a genomically stable isolate within the *S. aureus* population, while still reflecting the species’ capacity for ongoing microevolution through sporadic acquisition of accessory functions. This balance between conservation and variability is consistent with broader pangenome dynamics and reinforces the role of rare genes in shaping isolate-level genomic diversity.

### 3.6. Pangenome Openness, Genomic Similarity, and Distribution of Core and Accessory Genes Across Staphylococcus aureus Genomes

**Figure 6:**
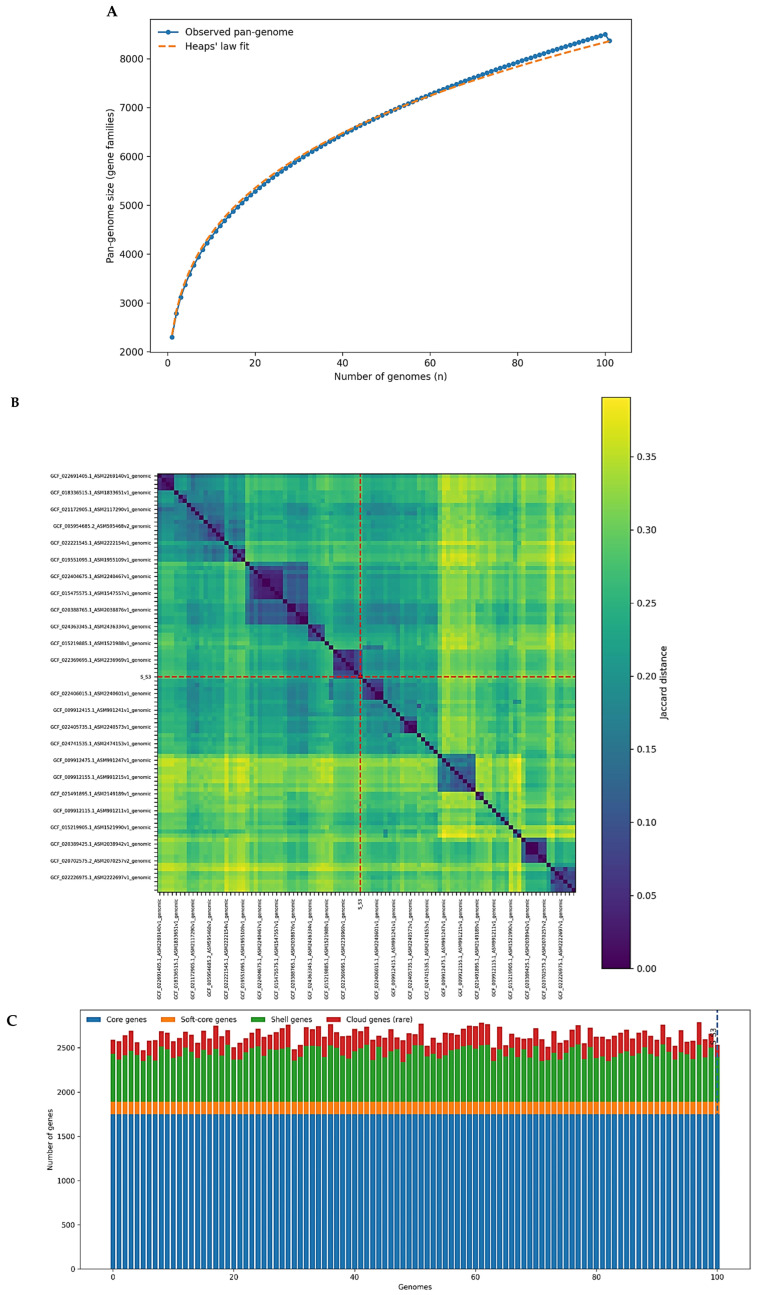
Pangenome openness, genomic similarity, and distribution of core and accessory genes across *Staphylococcus aureus* genomes. (**A**) Heaps’ law modeling of pangenome expansion based on the sequential incorporation of 100 *S. aureus* genomes. The observed pangenome size (points/solid line) is fitted to Heaps’ law (dashed line), yielding parameters k = 2346.55 and γ = 0.275. The γ value well below 1 indicates a clearly open pangenome, in which the addition of new genomes is expected to continuously introduce novel gene families, even at advanced sampling depths. (**B**) Heatmap of pairwise Jaccard distances calculated from gene presence–absence profiles, reflecting genomic similarity among the analyzed genomes. Lower values (cooler colors) indicate higher similarity, whereas higher values (warmer colors) reflect increased divergence driven primarily by differences in accessory gene content. Block-like patterns along the diagonal reveal clusters of closely related genomes, while the overall distribution supports substantial accessory-driven heterogeneity across the population. The position of the S_S3 isolate is highlighted, showing intermediate genomic similarity relative to the dataset. (**C**) Distribution of gene categories per genome, illustrating the relative contributions of core, soft core, shell, and cloud (rare) gene families. The blue dashed line indicates the position of the S_S3 isolate. Core genes are consistently conserved across all genomes, forming a stable genomic backbone, whereas shell and cloud genes display marked variability among isolates and account for most inter-genome differences in total gene content. The S_S3 isolate exhibits complete retention of core genes and a limited but detectable fraction of cloud genes, consistent with its placement in the genomic similarity landscape and the open pangenome structure inferred for *S. aureus*.

The openness and evolutionary dynamics of the *Staphylococcus aureus* pangenome were quantitatively assessed using Heaps’ law modeling applied to the cumulative gene family expansion across the 100 analyzed genomes (Figure 6A). The fitted model yielded a scaling parameter γ = 0.275 and a proportionality constant k = 2346.55, indicating a clearly open pangenome structure. The γ value well below 1 demonstrates that the incorporation of additional genomes is expected to continuously introduce new gene families, even as sampling depth increases. This behavior is consistent with sustained gene discovery observed in the empirical pangenome accumulation curves and supports ongoing genomic diversification driven by accessory gene flux.

To complement the cluster level organization observed in Figure 3A,B, we quantified genome-wide pairwise gene content dissimilarity using Jaccard distances derived from the presence–absence matrix (Figure 6B). The heatmap reveals a heterogeneous but structured distribution of distances across the dataset, with most genome pairs exhibiting moderate Jaccard distances, indicative of a shared conserved backbone complemented by variable accessory gene content. The presence of distinct blocks of lower dissimilarity along the diagonal suggests the existence of closely related genomic clusters, whereas higher distance regions reflect more divergent accessory gene repertoires. The S_S3 isolate shows intermediate Jaccard distances relative to most genomes, placing it within the central diversity range of the population rather than as an outlier. This observation is consistent with the absence of missing core genes and the relatively small number of isolate-specific genes identified in S_S3, while still indicating variation within the accessory genome. The distribution of pairwise Jaccard distances further confirmed the absence of redundancy, with the majority of genome, pairs showing moderate dissimilarity and only a small fraction of highly similar pairs (39 out of 4950; 0.78%) exhibiting distances below 0.05 (Supplementary Figure S1). This pattern supports the representativeness of the dataset and indicates that the random sampling strategy did not lead to overrepresentation of closely related genomes.

To further resolve how gene categories contribute to genome level composition, we examined the distribution of Roary-defined core (99–100% presence), soft core, shell, and cloud genes across individual genomes (Figure 6C). Across the dataset, each genome harbors a highly consistent number of Roary core genes, reflecting strong conservation of essential biological functions throughout the species. Within this fraction, the strict core, defined as gene families present in 100% of the analyzed genomes, represents an even more conserved subset. Strict core genes include housekeeping and core metabolic functions such as gfo, menH, dhaL, dhaM, tenA, sspP, and ribosomal components, underscoring the exceptional stability of the conserved genomic backbone of *Staphylococcus aureus*.

In contrast, the number of shell and cloud genes varies substantially among genomes, accounting for most of the observed inter-genome differences in total gene counts. Shell genes, present in an intermediate fraction of genomes, form a sizeable and flexible component of the pangenome, whereas cloud genes, detected in only one or very few genomes, represent rare, low-frequency elements. Several cloud gene families (e.g., group_916, group_918, kdpA_3, rplK_2, rplK_3) occur in only a single genome (frequency = 0.01), highlighting the contribution of sporadic gene acquisition events to genomic individuality. The S_S3 genome conforms to this overall pattern, combining full retention of core genes with a modest number of clouds associated with gene families, consistent with its placement in the Jaccard similarity landscape.

Taken together, the concordant evidence from Heaps’ law modeling, Jaccard distance analysis, and genome level gene category composition demonstrates that *S. aureus* exhibits a strongly open pangenome characterized by a stable core and a highly dynamic accessory compartment. The interplay between conserved functions and variable shell and cloud genes underlies both the population-wide genomic plasticity and the emergence of isolate-specific traits, reinforcing the role of accessory gene turnover as a key driver of microevolution within this species.

## 4. Discussion

The present study provides a detailed and integrative characterization of the *Staphylococcus aureus* pangenome by combining a newly sequenced environmental isolate obtained from pigeon droppings in a hospital-adjacent setting with a uniformly annotated collection of 100 reference genomes. By applying a standardized analytical framework based on gene presence and absence matrices, genome accumulation dynamics, gene frequency spectra, Jaccard distance-based similarity, and isolate-specific gene content analysis, our findings offer a coherent view of the genomic architecture and plasticity of *S. aureus* within a One Health perspective.

The observed pangenome size of 8366 distinct gene families and its persistent expansion throughout genome incorporation provide robust evidence that *S. aureus* possesses a clearly open pangenome. From a public health perspective, this genomic architecture implies a continuous capacity for the acquisition of novel accessory genes, including antimicrobial resistance and virulence determinants. Such evolutionary flexibility reinforces the need for sustained genomic surveillance across human, animal, and environmental reservoirs, as new genetic elements emerging in environmental niches may eventually enter clinical populations within interconnected One Health systems. These findings are fully consistent with previous comparative genomic studies that examined isolates from clinical, livestock, wildlife, and environmental sources, all of which converged on the classification of *S. aureus* as an open pangenome species [14,15,16,17,18]. Importantly, the convergence of terminal pangenome size across multiple random permutations demonstrates that the inferred gene repertoire is robust to genome ordering effects, supporting the biological authenticity of the observed diversity rather than analytical artifacts. Similar robustness of pangenome expansion has been reported in population genomic studies spanning diverse hosts and ecological contexts [14,19].

In contrast to the continuously expanding pangenome, the core genome exhibited a clear stabilization pattern. Under the 99 to 100 percent presence threshold, the core genome converged to approximately 1757 gene families, while the strictly conserved fraction present in all genomes comprised 1651 genes. This distinction highlights that a subset of genes is nearly universal yet not absolutely fixed across all genomes, reflecting subtle but biologically meaningful variation within the conserved backbone. Comparable ranges of core genome size have been reported in the literature, with substantial variability depending on population structure and sampling breadth, ranging from fewer than 600 genes in lineage-focused wildlife studies to over 2000 genes in datasets with narrower ecological scope [20,21,22]. These differences reinforce the notion that core genome size is not a fixed species-level attribute but a function of sampling diversity and ecological heterogeneity.

The gene frequency spectrum observed in this study further supports the interpretation of pronounced genomic plasticity in *S. aureus*. The dominance of low-frequency and unique gene families, with more than one thousand genes detected in only a single genome, defines a substantial cloud genome that constitutes the largest component of the pangenome. This pattern mirrors observations from comparative analyses of environmental, wildlife, and multi-host datasets, which consistently report disproportionately large accessory gene pools relative to predominantly clinical collections [17,21,23]. Such rare genes are widely interpreted as the product of horizontal gene transfer, mobile genetic elements, and niche-specific selective pressures, collectively driving ongoing microevolution and ecological diversification.

The relatively modest size of the shell genome and its gradual frequency distribution suggest that gene retention in *S. aureus* operates along a continuum rather than discrete categorical boundaries. Shell genes likely encode adaptive traits that confer selective advantages in specific ecological, or host-associated contexts without being universally required. Similar interpretations have been proposed in studies of livestock and wildlife-associated *S. aureus*, where shell genes were shown to underpin lineage-specific virulence patterns, antimicrobial resistance profiles, and host adaptation [16,18,22]. Several genes located within the shell genome in previous comparative genomic studies include loci associated with virulence regulation, antimicrobial resistance, stress tolerance, and host interaction. These intermediate frequency genes may therefore contribute to ecological specialization, enabling certain lineages to persist in environmental reservoirs while retaining the potential to participate in clinical transmission cycles.

At the population level, Jaccard distance-based analyses revealed clear gene content-driven structuring across the dataset. Both principal component analysis and hierarchical clustering converged on the presence of multiple coherent genomic clusters, despite relatively constrained variation in total genome size. This finding underscores those differences in accessory gene composition, rather than large-scale genome expansion or contraction, are the principal drivers of population structure in *S. aureus*. While many previous studies relied primarily on phylogenetic or single-nucleotide polymorphism-based frameworks [14,24,25], explicit quantitative assessment of accessory genome similarity using Jaccard metrics has been infrequently applied. Our results therefore provide a valuable quantitative complement to existing phylogenomic evidence, demonstrating that accessory gene turnover alone is sufficient to generate structured genomic stratification. The strong concordance observed between accessory genome-based clustering and MLST-defined clonal complexes further reinforces that gene presence and absence patterns capture the established population structure in *S. aureus*.

Within this framework, the placement of the S_S3 environmental isolate within a defined genomic cluster rather than as an extreme outlier is particularly informative. S_S3 retained the complete core genome and contributed only a limited number of unique gene families, indicating strong genomic integration within the broader *S. aureus* population. This observation contrasts with the assumption that environmental, or wildlife-associated isolates necessarily occupy highly divergent genomic positions. Instead, it aligns with accumulating evidence of extensive genetic overlap and bidirectional exchange between clinical, livestock, and environmental populations [17,26,27].

The isolate-specific gene content of S_S3 further supports this interpretation. The identification among S_S3-exclusive genes of linA, encoding a lincosamide resistance determinant, and efeB, a deferrochelatase/peroxidase-related gene involved in iron metabolism and oxidative stress response, suggests niche-adaptive fine-tuning rather than large-scale genomic divergence. Importantly, the identification of isolate-specific genes is inherently dependent on sampling depth and dataset composition. As additional genomes are incorporated, particularly from underrepresented ecological niches, some genes classified as unique may be detected in other lineages and thus reclassified as part of the broader accessory genome. Therefore, the S_S3-specific genes identified here should be interpreted as dataset-specific, within the context of the analyzed genome collection rather than universally exclusive to this isolate. Similar patterns have been described in environmental and wildlife isolates, which frequently harbor resistance and stress response genes embedded within otherwise conserved genomic backgrounds [23,28]. The predominance of hypothetical proteins among isolate-specific genes is also consistent with previous pangenome studies, where low-frequency genes often lack functional annotation, reflecting both genuine biological novelty and current limitations of annotation pipelines [15,19].

The Heaps’ law parameter estimated in this study (γ = 0.275) quantitatively confirms the open nature of the *S. aureus* pangenome and closely aligns with values reported in multi-host and multi-lineage studies [16,17,18]. This low gamma value indicates that continued genome sequencing is expected to yield additional gene families, particularly from underrepresented ecological niches. Environmental isolates from non-host matrices and wildlife-associated isolates, which remain sparsely sampled in public databases, are therefore likely to contribute disproportionately to future pangenome expansion, as suggested by their higher accessory gene diversity reported in comparative studies [21,22].

Taken together, these findings reinforce a unifying view of *S. aureus* as a species characterized by a compact and highly conserved core genome overlaid by a vast, dynamic, and continuously remodeling accessory gene reservoir. This genomic architecture provides a mechanistic basis for the remarkable ecological versatility of *S. aureus*, enabling persistence across human, animal, and environmental settings while maintaining essential pathogenic potential. Importantly, the inclusion of a pigeon dropping-derived wildlife-associated isolation from a hospital-adjacent outdoor environment addresses a notable gap in the literature, as these interfaces remain poorly represented despite their epidemiological relevance.

An additional technical point concerns the standardized Prokka reannotation step used to harmonize gene calling across genomes before pangenome inference. Because gene presence/absence analyses are highly sensitive to annotation heterogeneity, public annotations generated under mixed conventions were not combined directly. Instead, all genomes had to yield standardized Prokka GFF3 outputs under a single frozen workflow. To better understand whether the annotation failures reflected a general workflow instability, we conducted an auxiliary audit using an independent sample of complete RefSeq genomes under the same computational environment. All tested genomes were successfully annotated and yielded valid GFF3 outputs. This indicates that the Prokka-based workflow itself was operationally stable for high-quality complete assemblies and suggests that the failures observed during large-scale preprocessing were more likely associated with input-specific characteristics of the broader original genome collection, including heterogeneity in assembly structure or other technical compatibility constraints that became relevant only at larger scale. Accordingly, the final dataset should be interpreted as a reproducible, diversity-aware reference panel for standardized comparative inference rather than as an exhaustive representation of all publicly available *Staphylococcus aureus* diversity. The use of a frozen inclusion list, uniform annotation, and explicit quality control criteria was intended to maximize methodological consistency and reproducibility across all downstream analyses.

Some limitations of this study should be acknowledged. Although the dataset integrates a newly sequenced environmental isolate with a substantial number of reference genomes, environmental and wildlife-associated isolates remain underrepresented relative to clinical genomes in public repositories, reflecting a broader bias in the genomic data currently available. An additional limitation is that the study centers on a single newly sequenced environmental isolation. Therefore, broader claims regarding wildlife-associated *Staphylococcus aureus* should be interpreted cautiously until additional genomes from comparable ecological settings are incorporated into similar comparative frameworks.

Another priority for future investigation is the direct comparison of environmental, and wildlife-associated isolates with patient-derived *S. aureus* genomes, particularly from the same hospital setting. Such analyses could help identify shared accessory determinants, resistance-associated loci, and lineage overlap between hospital-adjacent environmental reservoirs and clinical populations, thereby strengthening the translational relevance of One Health genomic surveillance. Furthermore, although the use of random sampling for genome selection was computationally tractable and compatible with the aims of the present study, it may not fully capture the complete spectrum of lineage diversity when compared with strategies based on objective clustering criteria, such as average nucleotide identity (ANI) or core genome phylogenetic reconstruction.

In addition, while the standardized annotation strategy was essential to ensure comparability across genomes, large-scale preprocessing of public genome collections may be affected by technical tractability constraints that are difficult to predict a priori from accession metadata alone. In this context, the identification of isolate-specific genes should be interpreted with caution, as such classifications may shift with increased genome sampling, improved representation of environmental diversity, and the adoption of more diversity-aware genome preselection together with expanded preprocessing and annotation audit strategies in future studies.

Accordingly, while the present analyses robustly capture gene content structure within the sampled population, additional targeted sequencing from hospital-adjacent outdoor environments and wildlife-associated urban sources, as well as more diversity-informed genome selection strategies, would further refine estimates of accessory gene diversity and pangenome openness. Functional interpretation of low-frequency and hypothetical genes also remains constrained by current annotation frameworks, highlighting the need for integrative approaches that combine transcriptomic data and experimental validation to better resolve the biological relevance of these elements.

Despite these considerations, the standardized analytical approach and the concordance of results across multiple complementary analyses support the robustness and biological relevance of the conclusions presented here, while also highlighting promising directions for expanded One Health-oriented genomic surveillance of *Staphylococcus aureus*.

## 5. Conclusions

This study provides a genome-wide, pangenome-based characterization of *Staphylococcus aureus* that integrates a newly sequenced environmental isolate recovered from pigeon droppings in a hospital-adjacent setting with a uniformly annotated collection of 100 publicly available reference genomes. By applying a standardized analytical framework centered on gene presence and absence patterns, accumulation dynamics, gene frequency spectra, Jaccard distance-based similarity, and isolate-specific gene content assessment, the study delivers a coherent and internally consistent depiction of the genomic architecture of *S. aureus* within the analyzed dataset.

The results demonstrate that *S. aureus* exhibits a clearly open pangenome, as evidenced by the persistent expansion of the gene repertoire with increasing genome sampling and by a Heaps’ law scaling parameter (γ = 0.275) well below unity. Despite this openness, the species maintains a compact and stable conserved backbone, with a core genome converging under a 99–100% presence threshold and a slightly smaller strictly conserved fraction present in all genomes. These findings confirm that extensive accessory gene turnover coexists with strong conservation of essential genomic functions.

Analysis of gene frequency distributions revealed that the pangenome is dominated by low-frequency and strain-restricted gene families, defining a large cloud genome that represents the principal source of genomic diversity. This observation also highlights the importance of future comparisons with patient-derived genomes, since low-frequency accessory genes may help clarify potential links between hospital-adjacent environmental reservoirs and strains relevant to human health. In contrast, the shell genome constitutes a more limited but structured intermediate layer, contributing disproportionately to genome differentiation. Together, these patterns indicate that genomic plasticity in *S. aureus* is primarily driven by rare and intermediate frequency genes rather than variation within the conserved core.

Gene content similarity analyses based on Jaccard distances further showed that the *S. aureus* population is organized into distinct genomic clusters shaped by accessory gene composition, even in the absence of major differences in total genome size. Both multivariate ordination and hierarchical clustering converged on this structure, underscoring the utility of gene presence and absence-based approaches for resolving population organization beyond sequence-based phylogenetic frameworks alone.

Within this population context, the newly sequenced environmental isolate S_S3 displayed complete retention of the conserved core genome and contributed only a limited number of isolate-specific gene families. Its placement within a defined genomic cluster, rather than as a divergent outlier, indicates close integration with established *S. aureus* gene content backgrounds. The small set of S_S3-specific genes, including a lincosamide resistance determinant and a gene associated with oxidative stress response, reflects modest accessory variation consistent with localized or niche associated adaptation rather than extensive genomic divergence.

Overall, the findings support a model in which *Staphylococcus aureus* is characterized by a stable conserved backbone overlaid by a large, dynamic, and continuously evolving accessory gene reservoir. Within the limits of the dataset analyzed, the environmental isolate S_S3 did not appear genomically exceptional but instead fit within the broader spectrum of *S. aureus* gene content diversity. This study therefore provides a reproducible, pangenome level framework for contextualizing environmental *S. aureus* isolates and establishes a foundation for future investigations incorporating broader ecological sampling to refine estimates of accessory gene diversity and pangenome dynamics under a One Health perspective.

## Data Availability

The genome sequence of isolate S_S3 is available at NCBI GenBank (BioProject PRJNA1415265; BioSample SAMN54927991). All processed datasets, scripts, and reproducibility files are openly available at the Open Science Framework (OSF) repository: https://doi.org/10.17605/OSF.IO/3C4B9.

## Supplementary Materials

The following supporting information can be downloaded at: https://www.mdpi.com/article/10.3390/microorganisms14040938/s1, Figure S1: Distribution of pairwise Jaccard distances; Table S1: Genome assembly statistics and quality assessment metrics for all analyzed genomes; Table S2: MLST sequence type (ST) and clonal complex (CC) assignments for all genomes included in the study.

## Abbreviations

The following abbreviations are used in this manuscript:

AMR	Antimicrobial resistance
CLSI	Clinical and Laboratory Standards Institute
GFF3	General Feature Format, version 3
MRSA	Methicillin-resistant *Staphylococcus aureus*
ESKAPE	*Enterococcus faecium*, *Staphylococcus aureus*, *Klebsiella pneumoniae*, *Acinetobacter baumannii*, *Pseudomonas aeruginosa* and *Enterobacter* spp.
PCA	Principal component analysis
PC1	Principal component 1
PC2	Principal component 2
