# Supplementary material for: Pangenome Architecture and Accessory Gene-Driven Population Structure of Staphylococcus aureus Revealed by a Hospital-Adjacent Environmental Isolate
Source: Microorganisms. 2026 Apr 21;14(4):938. doi: 10.3390/microorganisms14040938 (PMC13118437; doi:10.3390/microorganisms14040938)
Supplement: Supplementary file 1 [file microorganisms-14-00938-s001.zip › microorganisms-4156465-supplementary.pdf]

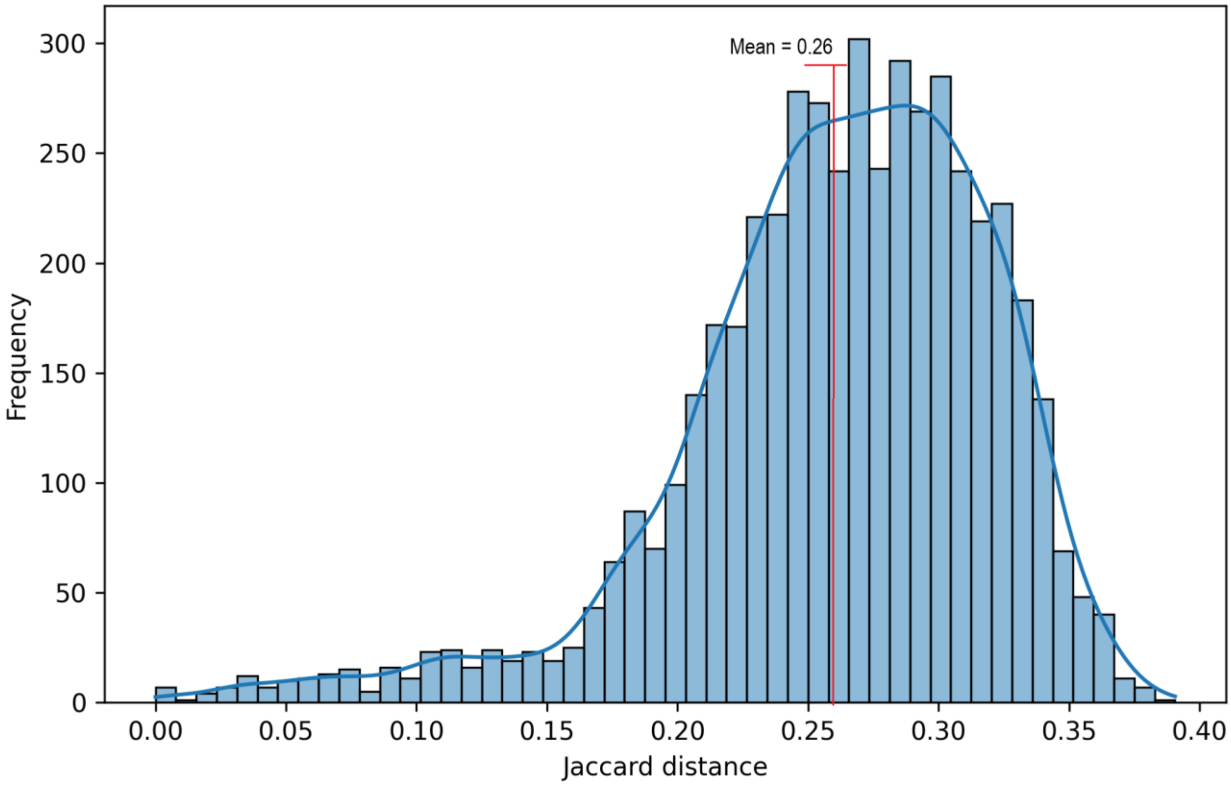

Figure S1. Distribution of pairwise Jaccard distances across the 100 Staphylococcus aureus genomes. The histogram shows the frequency of pairwise gene content dissimilarity values derived from the gene presence–absence matrix. The red vertical line indicates the mean Jaccard distance (0.26). The distribution spans a broad range (0.00–0.39) and is centered around moderate dissimilarity values. Only a small fraction of genome pairs exhibits very low dissimilarity (<0.05), indicating minimal redundancy and supporting the representativeness of the dataset.

Supplementary Table S1. Genome assembly statistics and quality assessment metrics for all analyzed genomes.

Genome	Genome_Size	GC	Contigs	N50	CheckM2_Completeness	CheckM2_Contamination	BUSCO Completeness
GCF_005954685.2_ASM595468v2	2847446	0.33	2	2820179	100	0.4	100
GCF_009912115.1_ASM991211v1	2760735	0.33	1	2760735	100	0.12	100
GCF_009912155.1_ASM991215v1	2883314	0.33	1	2883314	100	0.37	100
GCF_009912415.1_ASM991241v1	2712116	0.33	2	2709643	100	0.06	100
GCF_009912455.1_ASM991245v1	2672314	0.33	1	2672314	100	0.09	100
GCF_009912475.1_ASM991247v1	2948281	0.33	1	2948281	100	0.22	100
GCF_009912715.1_ASM991271v1	2896381	0.33	1	2896381	100	0.6	100
GCF_009912735.1_ASM991273v1	2823974	0.33	2	2798472	100	0.06	100
GCF_009913015.1_ASM991301v1	2897814	0.33	2	2870548	100	0.57	100
GCF_009913135.1_ASM991313v1	2890732	0.33	2	2863573	100	0.4	100
GCF_011032785.1_ASM1103278v1	2777910	0.33	2	2752113	100	0.07	100
GCF_013467165.1_ASM1346716v1	2853017	0.33	1	2853017	100	0.07	100
GCF_014696255.1_ASM1469625v2	2879954	0.33	1	2879954	100	0.09	100
GCF_015219885.1_ASM1521988v1	2917593	0.33	2	2888890	100	0.08	100
GCF_015219905.1_ASM1521990v1	2941588	0.33	2	2934806	100	0.88	100
GCF_015475575.1_ASM1547557v1	2877525	0.33	2	2874400	100	0.09	100
GCF_015774815.1_ASM1577481v1	2731537	0.33	1	2731537	100	0.08	100
GCF_015774855.1_ASM1577485v1	2738766	0.33	1	2738766	100	0.05	100
GCF_016591995.1_ASM1659199v1	2863321	0.33	2	2838668	100	0.34	100
GCF_016727585.1_ASM1672758v1	2776467	0.33	2	2756709	100	0.06	100
GCF_016889145.1_ASM1688914v1	2891871	0.33	3	2856112	100	0.08	99,1
GCF_016889945.1_ASM1688994v1	2888765	0.33	2	2856112	100	0.08	100
GCF_017161095.1_ASM1716109v1	2746973	0.33	2	2730873	100	0.03	100
GCF_018093065.1_ASM1809306v1	2803377	0.33	2	2766936	100	0.69	100
GCF_018336515.1_ASM1833651v1	2929593	0.33	2	2890366	100	0.33	100
GCF_018987325.1_ASM1898732v1	2832304	0.33	4	2809746	100	0.08	100
GCF_019334585.1_ASM1933458v1	2835652	0.33	1	2835652	100	0.15	100
GCF_019550945.1_ASM1955094v1	2956278	0.33	2	2931169	100	0.42	100
GCF_019551035.1_ASM1955103v1	2904018	0.33	1	2904018	100	0.39	100
GCF_019551095.1_ASM1955109v1	2972739	0.33	2	2947630	100	0.44	100

GCF_019551355.1_ASM1955135v1	2760709	0.33	2	2757377	100	0.08	100
GCF_019551375.1_ASM1955137v1	2917929	0.33	2	2893276	100	0.04	100
GCF_019915425.1_ASM1991542v1	2889511	0.33	1	2889511	100	0.12	100
GCF_019915585.1_ASM1991558v1	2926847	0.33	2	2888791	100	0.71	100
GCF_020034535.1_ASM2003453v1	2913602	0.33	3	2908126	100	0.19	100
GCF_020388335.1_ASM2038833v1	2757494	0.33	1	2757494	100	0.05	100
GCF_020388555.1_ASM2038855v1	2926152	0.33	5	2855863	100	0.21	100
GCF_020388715.1_ASM2038871v1	2745573	0.33	1	2745573	100	0.05	100
GCF_020388765.1_ASM2038876v1	2745575	0.33	1	2745575	100	0.05	100
GCF_020388925.1_ASM2038892v1	2952667	0.33	2	2919985	100	1.74	100
GCF_020389005.1_ASM2038900v1	2748827	0.33	1	2748827	100	0.07	100
GCF_020389325.1_ASM2038932v1	2719233	0.33	1	2719233	100	0.11	100
GCF_020389425.1_ASM2038942v1	2719235	0.33	1	2719235	100	0.11	100
GCF_020389445.1_ASM2038944v1	2719166	0.33	1	2719166	100	0.1	100
GCF_020389485.1_ASM2038948v1	2754401	0.33	1	2754401	100	0.05	100
GCF_020389525.1_ASM2038952v1	2828046	0.33	3	2754404	100	0.08	100
GCF_020702535.2_ASM2070253v2	2715164	0.33	2	2690509	100	0.11	100
GCF_020702575.2_ASM2070257v2	2741162	0.33	3	2716742	100	0.12	100
GCF_021172845.1_ASM2117284v1	2758520	0.33	2	2737790	100	0.14	100
GCF_021172905.1_ASM2117290v1	2780115	0.33	1	2780115	100	0.06	100
GCF_021390135.1_ASM2139013v1	2742047	0.33	2	2740225	100	0.05	100
GCF_021491715.1_ASM2149171v1	2782272	0.33	1	2782272	100	0.06	100
GCF_021491895.1_ASM2149189v1	2731384	0.33	1	2731384	100	0.13	100
GCF_022221525.1_ASM2222152v1	2849687	0.33	1	2849687	100	0.07	100
GCF_022221545.1_ASM2222154v1	2956826	0.33	2	2939569	100	0.11	100
GCF_022226975.1_ASM2222697v1	2700946	0.33	1	2700946	100	0.09	100
GCF_022369535.1_ASM2236953v1	2816536	0.33	2	2799207	100	0.05	100
GCF_022369595.1_ASM2236959v1	2896504	0.33	4	2863885	100	0.08	100
GCF_022369695.1_ASM2236969v1	2768180	0.33	2	2743526	100	0.05	100
GCF_022404575.1_ASM2240457v1	2843605	0.33	2	2816537	100	0.07	100
GCF_022404595.1_ASM2240459v1	2800541	0.33	2	2779850	100	0.09	100
GCF_022404635.1_ASM2240463v1	2775445	0.33	3	2725874	100	0.1	100
GCF_022404675.1_ASM2240467v1	2840252	0.33	3	2823979	100	0.04	100

GCF_022404735.1_ASM2240473v1	2739777	0.33	2	2737213	100	0.05	100
GCF_022404755.1_ASM2240475v1	2791775	0.33	2	2771054	100	0.09	100
GCF_022405075.1_ASM2240507v1	2868283	0.33	2	2823053	100	0.18	100
GCF_022405185.1_ASM2240518v1	2746469	0.33	2	2729778	100	0.04	100
GCF_022405235.1_ASM2240523v1	2756049	0.33	2	2735369	100	0.06	100
GCF_022405255.1_ASM2240525v1	2751661	0.33	2	2722643	100	0.05	100
GCF_022405355.1_ASM2240535v1	2950572	0.33	2	2923504	100	0.37	100
GCF_022405455.1_ASM2240545v1	2801355	0.33	2	2774090	100	0.15	100
GCF_022405535.1_ASM2240553v1	2750046	0.33	2	2747630	100	0.06	100
GCF_022405735.1_ASM2240573v1	2744744	0.33	1	2744744	100	0.03	100
GCF_022405795.1_ASM2240579v1	2735271	0.33	1	2735271	100	0.06	100
GCF_022405835.1_ASM2240583v1	2839488	0.33	1	2839488	100	0.14	100
GCF_022405895.1_ASM2240589v1	2824979	0.33	1	2824979	100	0.2	100
GCF_022406015.1_ASM2240601v1	2747195	0.33	1	2747195	100	0.06	100
GCF_022406055.1_ASM2240605v1	2746529	0.33	1	2746529	100	0.06	100
GCF_022406435.1_ASM2240643v1	2743582	0.33	1	2743582	100	0.03	100
GCF_022406675.1_ASM2240667v1	2923870	0.33	2	2884711	100	0.1	99,1
GCF_022691325.1_ASM2269132v1	2922786	0.33	2	2898133	100	0.12	100
GCF_022691405.1_ASM2269140v1	2844400	0.33	2	2819747	100	0.1	100
GCF_022693265.1_ASM2269326v1	2923261	0.33	3	2820042	100	0.12	100
GCF_022693325.1_ASM2269332v1	2922615	0.33	2	2897962	100	0.15	100
GCF_022699245.1_ASM2269924v1	2911979	0.33	3	2840066	100	0.12	100
GCF_022699265.1_ASM2269926v1	2911855	0.33	3	2843362	100	0.16	100
GCF_022870625.1_ASM2287062v1	2832381	0.33	1	2832381	100	0.14	100
GCF_023278065.1_ASM2327806v1	2697590	0.33	1	2697590	100	0.04	100
GCF_024172245.1_ASM2417224v1	2898303	0.33	3	2878495	100	0.09	100
GCF_024296825.1_ASM2429682v1	2697642	0.33	1	2697642	100	0.04	100
GCF_024296845.1_ASM2429684v1	2912407	0.33	1	2912407	100	0.41	100
GCF_024363325.1_ASM2436332v1	2857031	0.33	2	2835705	100	0.19	99,1
GCF_024363345.1_ASM2436334v1	2831391	0.33	2	2810065	100	0.16	100
GCF_024363385.1_ASM2436338v1	2824903	0.33	2	2803577	100	0.18	97,4
GCF_024363485.1_ASM2436348v1	2926742	0.33	4	2850449	100	0.03	100
GCF_024612055.1_ASM2461205v1	2741379	0.33	1	2741379	100	0.08	100

GCF_024741395.1_ASM2474139v1	2787288	0.33	1	2787288	100	0.05	100
GCF_024741495.1_ASM2474149v1	2744047	0.33	1	2744047	100	0.04	100
GCF_024741535.1_ASM2474153v1	2799824	0.33	1	2799824	100	0.02	100
S_S3	2775544	0.3273	25	534742	100	0.83	99,6

Table S2. MLST sequence type (ST) and clonal complex (CC) assignments for all genomes included in the study.

Genome	ST	CC
GCF_005954685.2_ASM595468v2	5	CC5
GCF_009912115.1_ASM991211v1	1027	Unassigned_CC
GCF_009912155.1_ASM991215v1	30	CC30
GCF_009912415.1_ASM991241v1	582	Unassigned_CC
GCF_009912455.1_ASM991245v1	45	CC45
GCF_009912475.1_ASM991247v1	34	Unassigned_CC
GCF_009912715.1_ASM991271v1	30	CC30
GCF_009912735.1_ASM991273v1	30	CC30
GCF_009913015.1_ASM991301v1	225	Unassigned_CC
GCF_009913135.1_ASM991313v1	225	Unassigned_CC
GCF_011032785.1_ASM1103278v1	6715	Unassigned_CC
GCF_013467165.1_ASM1346716v1	30	CC30
GCF_014696255.1_ASM1469625v2	789	Unassigned_CC
GCF_015219885.1_ASM1521988v1	2066	Unassigned_CC
GCF_015219905.1_ASM1521990v1	772	Unassigned_CC
GCF_015475575.1_ASM1547557v1	8	CC8
GCF_015774815.1_ASM1577481v1	133	Unassigned_CC
GCF_015774855.1_ASM1577485v1	49	Unassigned_CC
GCF_016591995.1_ASM1659199v1	5	CC5
GCF_016727585.1_ASM1672758v1	6	CC5
GCF_016889145.1_ASM1688914v1	8	CC8
GCF_016889945.1_ASM1688994v1	8	CC8
GCF_017161095.1_ASM1716109v1	97	CC97
GCF_018093065.1_ASM1809306v1	182	Unassigned_CC
GCF_018336515.1_ASM1833651v1	965	Unassigned_CC
GCF_018987325.1_ASM1898732v1	398	CC398
GCF_019334585.1_ASM1933458v1	8	CC8
GCF_019550945.1_ASM1955094v1	5	CC5
GCF_019551035.1_ASM1955103v1	5	CC5
GCF_019551095.1_ASM1955109v1	5	CC5
GCF_019551355.1_ASM1955135v1	72	CC72
GCF_019551375.1_ASM1955137v1	5	CC5
GCF_019915425.1_ASM1991542v1	30	CC30
GCF_019915585.1_ASM1991558v1	72	CC72
GCF_020034535.1_ASM2003453v1	45	CC45
GCF_020388335.1_ASM2038833v1	59	Unassigned_CC
GCF_020388555.1_ASM2038855v1	8	CC8
GCF_020388715.1_ASM2038871v1	8	CC8
GCF_020388765.1_ASM2038876v1	8	CC8
GCF_020388925.1_ASM2038892v1	1708	Unassigned_CC

GCF_020389005.1_ASM2038900v1	7	CC7
GCF_020389325.1_ASM2038932v1	398	CC398
GCF_020389425.1_ASM2038942v1	398	CC398
GCF_020389445.1_ASM2038944v1	398	CC398
GCF_020389485.1_ASM2038948v1	45	CC45
GCF_020389525.1_ASM2038952v1	45	CC45
GCF_020702535.2_ASM2070253v2	398	CC398
GCF_020702575.2_ASM2070257v2	398	CC398
GCF_021172845.1_ASM2117284v1	45	CC45
GCF_021172905.1_ASM2117290v1	5	CC5
GCF_021390135.1_ASM2139013v1	8	CC8
GCF_021491715.1_ASM2149171v1	7358	Unassigned_CC
GCF_021491895.1_ASM2149189v1	152	Unassigned_CC
GCF_022221525.1_ASM2222152v1	3686	Unassigned_CC
GCF_022221545.1_ASM2222154v1	5	CC5
GCF_022226975.1_ASM2222697v1	45	CC45
GCF_022369535.1_ASM2236953v1	25	Unassigned_CC
GCF_022369595.1_ASM2236959v1	8	CC8
GCF_022369695.1_ASM2236969v1	188	CC188
GCF_022404575.1_ASM2240457v1	8	CC8
GCF_022404595.1_ASM2240459v1	188	CC188
GCF_022404635.1_ASM2240463v1	45	CC45
GCF_022404675.1_ASM2240467v1	8	CC8
GCF_022404735.1_ASM2240473v1	188	CC188
GCF_022404755.1_ASM2240475v1	188	CC188
GCF_022405075.1_ASM2240507v1	51	Unassigned_CC
GCF_022405185.1_ASM2240518v1	9	CC8
GCF_022405235.1_ASM2240523v1	188	CC188
GCF_022405255.1_ASM2240525v1	5	CC5
GCF_022405355.1_ASM2240535v1	8	CC8
GCF_022405455.1_ASM2240545v1	5	CC5
GCF_022405535.1_ASM2240553v1	72	CC72
GCF_022405735.1_ASM2240573v1	97	CC97
GCF_022405795.1_ASM2240579v1	188	CC188
GCF_022405835.1_ASM2240583v1	7317	Unassigned_CC
GCF_022405895.1_ASM2240589v1	72	CC72
GCF_022406015.1_ASM2240601v1	72	CC72
GCF_022406055.1_ASM2240605v1	72	CC72
GCF_022406435.1_ASM2240643v1	97	CC97
GCF_022406675.1_ASM2240667v1	9	CC8
GCF_022691325.1_ASM2269132v1	5	CC5
GCF_022691405.1_ASM2269140v1	5	CC5
GCF_022693265.1_ASM2269326v1	5	CC5
GCF_022693325.1_ASM2269332v1	5	CC5
GCF_022699245.1_ASM2269924v1	5	CC5
GCF_022699265.1_ASM2269926v1	5	CC5
GCF_022870625.1_ASM2287062v1	105	Unassigned_CC

GCF_023278065.1_ASM2327806v1	8	CC8
GCF_024172245.1_ASM2417224v1	8	CC8
GCF_024296825.1_ASM2429682v1	8	CC8
GCF_024296845.1_ASM2429684v1	30	CC30
GCF_024363325.1_ASM2436332v1	1	CC1
GCF_024363345.1_ASM2436334v1	1	CC1
GCF_024363385.1_ASM2436338v1	1	CC1
GCF_024363485.1_ASM2436348v1	1	CC1
GCF_024612055.1_ASM2461205v1	1633	Unassigned_CC
GCF_024741395.1_ASM2474139v1	7752	Unassigned_CC
GCF_024741495.1_ASM2474149v1	7747	Unassigned_CC
GCF_024741535.1_ASM2474153v1	7745	Unassigned_CC
S_S3	188	CC188

CC assignments were inferred from PubMLST and eBURST based definitions. Sequence types without a defined clonal complex were classified as Unassigned_CC.
